# Saudi nursing students’ pain management knowledge and attitudes

**DOI:** 10.1002/nop2.570

**Published:** 2020-08-11

**Authors:** Emad Shdaifat, Noha Al‐Shdayfat, Abdallah Sudqi

**Affiliations:** ^1^ Department of Community Health Nursing College of Nursing Imam Abdulrahman Bin Faisal University City Dammam Saudi Arabia; ^2^ Community and Mental Health Nursing Department Faculty of Nursing Al al‐Bayt University Mafraq Jordan

**Keywords:** attitude, knowledge, pain management, nursing student

## Abstract

**Aim:**

To evaluate the level and identify predictors of nursing students’ knowledge and attitudes of pain management.

**Design:**

A cross‐sectional design was used to analyse nursing students’ knowledge and attitudes about pain management, with the Knowledge and Attitude Survey Regarding Pain (KASRP).

**Methods:**

A self‐administered questionnaire was used to collect data from a convenient sample of Saudi nursing students. A total of 193 nursing students from a nursing school at a Saudi university completed the questionnaires.

**Results:**

The student mean knowledge of pain management was 42.6 (*SD* 9.1) . The items answered correctly most frequently concerned pain medication and administration. On the other hand, the most commonly incorrect items were mainly related to assessment and pharmacological interventions. Logistic regression analysis found that gender was significantly associated with level of knowledge and attitudes regarding pain management.

## 1 INTRODUCTION

There is an increasing global acknowledgement of the importance of pain assessment that is increasingly recognized as a serious worldwide public health concern. Pain is an undesirable feeling related to tissue impairment and a cause for requiring medical services. Feeling pain has a undesirable effect on the quality of life and satisfaction level of patients, with increasing knowledge and perceptions of pain management and ways to alleviate symptoms and effects worldwide (Glowacki, 2015; Johnson, 2019). In recent years, there has been increasing interest in pain management as a vital part of medical treatment and nurses have a significant role in the evaluation and handling of pain events as they spend the most time liaising with patients in bedside care provision (de Araujo & Romero, 2015; Tompkins, Hobelmann, & Compton, 2017). Nurses can have a substantial impact on controlling pain if they have sufficient knowledge and positive perceptions towards pain management, but nurses consistently under‐estimate pain and deliver sub‐optimal pain control and treatment worldwide (Yava et al., 2013).

This is implicitly due to inadequate knowledge about pain management. Nursing students enter nursing programmes with a misunderstanding of pain and consequently continue to hold these erroneous concepts regardless of subsequent training and experience (Goldberg & McGee, 2011). Thus, a lack of training and misconceptions about pain management prevent nurses from providing appropriate treatment (Chen, Tsoy, Upadhye, & Chan, 2018; Germossa, Sjetne, & Hellesø, 2018). Some studies have focused specifically on pain management skills and knowledge among nurses (Gatchel, McGeary, McGeary, & Lippe, 2014; Umuhoza, Chironda, Katende, & Mukeshimana, 2019).

Research has consistently shown that pain profoundly influences patients’ physical and psychological status and thus has broader impacts on patients’ families and communities. Consequently, the theory‐practice gap (i.e. the poor knowledge and attitudes towards pain management among nurses) results in inadequate pain assessment and treatment in health systems (Department of Health & Human Services, 2019). The absence of international guidelines on pain management in medical and nursing programmes reduces the ability of healthcare professionals to evaluate and respond to patient pain (Department of Health & Human Services, 2019; Tabije‐Ebuen, 2018).

Nurses are at the front‐line of dealing with patients’ pain, through assessing and managing patients’ conditions and preferences on a frequent basis. However, despite many recommendations provided by the International Association for Pain Management and other bodies, it has been widely reported that undergraduate nursing curricula do not have adequate consideration of pain management (Burke, 2019; Latchman, 2014). Much uncertainty still exists about the pain as an unpleasant feeling for patients and it is a complicated experience in healthcare settings, which is not well detected or managed (Sherrill, 2013). Despite many empirical studies, advanced technological aides and pain management training workshops, pain treatment remains a challenge for health workers (Lewthwaite et al., 2011).

To find out the efficiency of current pain education on the knowledge, attitudes and perceptions of nursing students, valid measurement tools are required to assess and quantify these domains. This study thus undertakes to determine the level and predictors of knowledge and attitudes of nursing students towards pain management. The significance of this study is that its results can contribute to the literature about the level of student awareness and attitudes regarding pain management, which can help planners in the future to improve the quality of services provided for patients through better evaluation and treatment, thus improving patient health outcomes and satisfaction, providing optimum quality of care via evidence‐based practice (EBP).

## 2 METHODS

### 2.1 Setting

A cross‐sectional study was conducted at a college of nursing in a university in the Eastern Region of Saudi Arabia from March to May 2017. The college delivers a bachelor's degree in nursing and offers a bridging programme as well.

### 2.2 Sampling and sampling criteria

The population sample comprised all undergraduate and bridging students at the nursing college during the study period. Convenience sampling was used to collect data from the students during the study period. Using the online software Sampsize (http://sampsize.sourceforge.net/iface/s3.html), the calculated sample size was 163, through the following parameters (precision 5%, prevalence 50%, CL 95% and population 282). Nursing students who were studying in our school through March to May 2017 were eligible to take part in the study. We excluded students who did not attend pharmacological courses or who joined pain management workshops.

### 2.3 Survey instrument

The Knowledge and Attitudes Survey Regarding Pain (KASRP) was used to evaluate the knowledge and attitudes of nursing students towards pain management. The scale, originally developed by Ferrell and McCaffery in 1987, enables students to self‐report their responses to items designed to assay their knowledge and attitudes concerning pain and pain management (Ung, Salamonson, Hu, & Gallego, 2016). The English version of the scale was used, as English is the medium of instruction in the University and in the Saudi healthcare sector.

The KASRP is a self‐administered scale which includes 39 questions. The first 22 items are true or false questions. Questions 23–37 are multiple‐choice ones, and finally, questions 38–39 are case study questions. The tool has Cronbach's alpha of 0.70 and test–retest reliability of 0.80. The scoring for the correct answers was reported as percentages (Duke, Haas, Yarbrough, & Northam, 2013; Ung et al., 2016). The total scores ranged from 0–41, with higher scores indicating a higher degree of knowledge and attitudes regarding pain management. The scores are categorized into three categories: poor (<50%), fair (50–75) and good (>75). It should be noted that a very advanced level of prose is used in KASRP, which may be difficult even for native speaking nursing professionals to immediately comprehend, as may be judged from the examples quoted in the Results section below, which has methodological implications.

### 2.4 Ethical consideration

Research Ethics Committee approval was obtained from the Institutional Review Board (IRB) at the university where the research was carried out. The study protocol and questionnaire were approved prior to conducting any participant recruitment. At the outset, students were given a comprehensive Participant Information Sheet which, in addition to verbal communication, explained in full the voluntary nature of participation and the right to withdraw from the study at any time without giving a reason, without this affecting students’ education or statutory rights. It also explained the research purpose, significance and benefits.

### 2.5 Data collection

Data were collected during the teaching hours at the university. Thirty minutes were allocated to students to answer all the questions.

### 2.6 Data analysis

The analysis of data was done using SPSS version 20. Descriptive statistics and frequency distributions were performed to describe the baseline characteristics of study participants and to determine their level of knowledge and attitudes. Continuous data were assessed for normality, and chi‐square test was used to evaluate the association between demographic variables and level of pain management knowledge and attitudes. Binary logistic regression analysis was carried out to explore the independent predictor variables. A *p*‐value of <.05 was considered statistically significant.

## 3 RESULTS

### 3.1 Demographic characteristics

The response rate was 68.4%, and students’ mean age was 24.3 years (*SD* 4.6). 52.3% were male, 58.5% were single, and 72.8% were undergraduate students (thus, 27.2% were bridging students) (Table 1).

**Table 1:** Demographic characteristics of the participants (*n* = 193)

	Mean (*SD*)	Frequency	Percentage
Age	24.3 (4.6)		
Gender	Male	101	52.3
	Female	92	47.7
Marital status	Single	113	58.5
	Married	77	39.9
Education level	Undergraduate	132	72.8%
	Bridging	61	27.2%

### 3.2 Pain management knowledge and attitudes

The mean knowledge on pain management was 42.6 (*SD* 9.1). According to scoring criteria, 81% of nurses got poor scores and 19% got fair scores. Table 2 shows the relationship between proportion of demographic characteristics according to knowledge and attitude level. Chi‐square for gender shows that there is a significant difference between the knowledge and attitude of students and gender (*p* = .002). No significant association was found between knowledge and attitude score and marital status, educational level and age.

**Table 2:** Proportion of demographic characteristics according to knowledge and attitude level (*n* = 193)

	Poor	Fair	Sig.
Gender			
Male	90 (89.1%)	11 (10.9%)	0.002
Female	66 (71.7%)	26 (28.3%)	
Marital status			
Single	92 (81.4%)	21 (18.6%)	0.806
Married	64 (80.0%)	16 (20.0)	
Education level			
Undergraduate	103 (78.0%)	29 (22.0%)	0.146
Bridging	53 (86.9%)	8 (13.1%)	
Age			
<22	50 (76.9%)	15 (23.1%)	0.952
≥22	106 (82.8%)	22 (17.2%)	

Table 3 shows that the statements with the most correct responses were the following:

- “Sedation assessment is recommended during opioid pain management because excessive sedation precedes opioid‐induced respiratory depression” (over 75%).
- “After an initial dose of opioid analgesic is given, subsequent doses should be adjusted in accordance with the individual patient's response” (73.7%).
- “Narcotic/opioid addiction is defined as a chronic neurobiological disease, characterized by behaviours that include one or more of the following: impaired control over drug use, compulsive use, continued use despite harm and craving” (70.6%).

**Table 3:** Ten questions with the highest percentage answered correctly

Items	Corrected response	
	No.	%
Sedation assessment is recommended during opioid pain management because excessive sedation precedes opioid‐induced respiratory depression	172	75.4
After an initial dose of opioid analgesic is given, subsequent doses should be adjusted in accordance with the individual patient's response	168	73.7
Narcotic/opioid addiction is defined as a chronic neurobiological disease, characterized by behaviours that include one or more of the following: impaired control over drug use, compulsive use, continued use despite harm and craving	161	70.6
Patient's spiritual beliefs may lead them to think pain and suffering are necessary	155	68.0
The term “equianalgesia” means approximately equal analgesia and is used when referring to the doses of various analgesics that provide approximately the same amount of pain relief.	148	64.9
Combining analgesics that work by different mechanisms (e.g. combining an NSAID with an opioid) may result in better pain control with fewer side effects than using a single analgesic agent	142	62.3
Aspirin and other non‐steroidal anti‐inflammatory agents are not effective analgesics for painful bone metastases	130	57.0
The time to peak effect for morphine given IV is (15 min)	127	55.7
The recommended route of administration of opioid analgesics for patients with brief, severe pain of sudden onset, such as trauma or post‐operative pain, is (intravenous)	126	55.3
Which of the following analgesic medications is considered the drug of choice for the treatment of prolonged moderate‐to‐severe pain for cancer patients? (morphine)	125	54.8

The number of correct answers for other statements among the top ten *correctly* answered items ranged from 68.0%–54.8%.

Table 4 shows that the statements with the fewest correct responses were the following:

- “Received morphine 2 mg IV. Half‐hourly pain ratings following the injection ranged from 6–8, and he had no clinically significant respiratory depression, sedation or other untoward side effects. He has identified 2/10 as an acceptable level of pain relief. His physician's order for analgesia is morphine IV 1–3 mg q1h PRN pain relief” (8.3%).
- “The recommended route of administration of opioid analgesics for patients with persistent cancer‐related pain is (oral)” (8.3%).

**Table 4:** Ten questions with the lowest percentage answered correctly

Items	Corrected response	
	No.	%
If the source of the patient's pain is unknown, opioids should not be used during the pain evaluation period, as this could mask the ability to correctly diagnose the cause of pain	71	31.1
The time to peak effect for morphine given orally is (1–2 min)	69	30.3
How likely it is that patients who develop pain already have an alcohol and/or drug abuse problem? (5%–15%)	52	22.8
Following abrupt discontinuation of an opioid, physical dependence is manifested by the following: (sweating, yawning, diarrhoea and agitation with patients when the opioid is abruptly discontinued)	43	18.9
Patient B: Robert is 25 years old and this is his first day following abdominal surgery. As you enter his room, he is lying quietly in bed and grimaces as he turns in bed. Your assessment reveals the following information: BP = 120/80; HR = 80; R = 18; on a scale of 0 to 10 (0 = no pain/discomfort, 10 = worst pain/discomfort) he rates his pain as 8	43	18.9
Patient A: Andrew is 25 years old and this is his first day following abdominal surgery. As you enter his room, he smiles at you and continues talking and joking with his visitor. Your assessment reveals the following information: BP = 120/80; HR = 80; R = 18; on a scale of 0 to 10 (0 = no pain/discomfort, 10 = worst pain/discomfort) he rates his pain as 8	36	15.8
A patient with persistent cancer pain has been receiving daily opioid analgesics for 2 months. Yesterday, the patient was receiving morphine 200 mg/hr intravenously. Today, he has been receiving 250 mg/hr intravenously. The likelihood of the patient developing clinically significant respiratory depression in the absence of new comorbidity is (<1%)	25	11.0
Your assessment, above, is made 2 hrs after he received morphine 2 mg IV. Half‐hourly pain ratings following the injection ranged from 6–8, and he had no clinically significant respiratory depression, sedation or other untoward side effects. He has identified 2/10 as an acceptable level of pain relief. His physician's order for analgesia is “morphine IV 1–3 mg q1h PRN pain relief.” Check the action you will take at this time: (administer morphine 3 mg IV now)	23	10.1
The recommended route of administration of opioid analgesics for patients with persistent cancer‐related pain is (oral)	19	8.3
Your assessment, above, is made 2 hrs after he received morphine 2 mg IV. Half‐hourly pain ratings following the injection ranged from 6–8, and he had no clinically significant respiratory depression, sedation or other untoward side effects. He has identified 2/10 as an acceptable level of pain relief. His physician's order for analgesia is “morphine IV 1–3 mg q1h PRN pain relief.” Check the action you will take at this time (administer morphine 3 mg IV now)	19	8.3

The number of correct answers for other statements among the top ten *incorrectly* answered items ranged from 10.1%–31.1%.

Binary logistic regression was used for four variables (age, gender, education level and marital status) as predictors of knowledge and attitudes among students. The Omnibus Test of Model coefficient table indicates that when four variables are considered together, the model or equation is significant (*X* ^2^ = 13.3, *df* = 4, *N* = 193, *p* = .01). Nagelkerke's *R* ^2^ = .10 indicates a weak relationship between prediction and gender (82.9%). The Wild criterion demonstrates that only gender made a significant contribution to prediction (*p* = .002) (Table 5).

**Table 5:** Logistic regression analysis of variables associated with knowledge and attitude of student

	B (*SE*)	95% Cl for odds ratio		
		Odds ratio	Lower	Upper
Constant	−0.58 (0.85)			
Age	0.16 (0.43)	1.18	0.51	2.74
Gender*	−1.23 (0.40)	0.29	0.13	0.64
Marital status	−0.30 (0.43)	0.74	0.32	1.72
Education level	−0.21 (0.55)	0.81	0.27	2.38

Note

*R* ^2^ = .98 (Hosmer & Lemeshow), 0.06 (cox & Snell), 0.10 (Nagelkerke), Model *X* ^2^ (4) = 13.31, *p* = .002.

^*^ *p* < .01.

## 4 DISCUSSION

This study investigated Saudi nursing students’ knowledge and attitudes towards pain management and found them to be generally inadequate. Approximately three‐quarters of the nursing students correctly answered a question regarding sedation assessment (“*Sedation assessment is recommended during opioid pain management because excessive sedation precedes opioid‐induced respiratory depression*”), but all other items were answered correctly by fewer than 75% of participants.

The findings of the current study showed that there is insufficient knowledge among nursing students with regard to pain medications and assessment, as low percentages of correct answers were reported. This finding is congruent with previous studies (Al‐Khawaldeh, Al‐Hussami, & Darawad, 2013; Goodrich, 2006; Lewthwaite et al., 2011; Rahimi‐Madiseh, Tavakol, & Dennick, 2010). One notable finding is that only 54.8% of the participants knew that morphine is considered the drug of choice for the treatment of prolonged moderate‐to‐severe pain for cancer patients. Our results agree with the results obtained in a study carried out in Jordan, where almost half of the respondents correctly responded to the same item (54.5%) (Al Omari, 2016). Thus, it is critically important to enrich undergraduate nursing students’ pharmacological knowledge regarding pain management (Al Omari, 2016; Al‐Khawaldeh et al., 2013; Gadallah, Hassan, & Shargawy, 2017).

The level of education might be intuitively expected to be significantly associated with the knowledge and attitudes of the students in terms of pain management, and this is affirmed by some empirical investigations (Duke et al., 2013; Ekim & Ocakcı, 2013; Lewthwaite et al., 2011). This reflects that seniors are expected to have more theoretical and clinical courses than juniors, as well as more clinical experience. However, the results of the current study support previous investigations which reached the opposite conclusion: that educational level is not significantly associated with students’ knowledge and attitudes (Craig, 2014; Shakya & Shakya, 2016). At any rate, it is crucial for the nursing education curriculum to be updated and revised to ensure that it contributes to enrich the students’ knowledge as they move from one level of education to the next.

The items answered correctly most frequently focus on pain medication and administration, while the most commonly incorrect items were mainly related to assessment and pharmacological intervention. This result is consistent with studies declaring that current nursing education does not adequately emphasize students’ abilities to assess their patients’ needs and intervene properly in response (Alnajar, Darawad, Alshahwan, & Samarkandi, 2019; Manwere, Chipfuwa, Mukwamba, & Chironda, 2015).

Gender is significantly associated with students’ knowledge of pain management, with female students having better knowledge than males. Moreover, gender was found to be a predictor of knowledge and attitudes among students. This result is concurrent with previous research findings (Greenberger, Reches, & Riba, 2006), specifically relating to the behavioural dimension of pain assessment. However, in other studies, gender was not found to exert any significant effect on undergraduate nursing students’ knowledge of pain management (Gadallah et al., 2017). As female student nurses are generally more empathetic than their male counterparts (Ouzouni & Nakakis, 2012), it can be inferred that they may pay more attention to pain management information.

## 5 CONCLUSION

The present study was designed to determine the level and predictors of knowledge and attitudes of the nursing students towards pain management. The most obvious finding to emerge from this study is that the students’ mean knowledge of pain management was 42.6%. The items answered correctly most frequently concerned pain medication and administration. Furthermore, the most commonly incorrect items were mainly related to assessment and pharmacological interventions. Regression analysis found that gender was significantly associated with the level of knowledge and attitudes about pain management.

The most obvious implication of this study is the low percentages of Saudi nursing students’ knowledge regarding pain management treatment. Consequently, we recommend strengthening the current pharmacology curriculum for nursing students, which is clearly inadequate in terms of imparting knowledge of pain management treatments.

The results of this study contribute to the literature about the level of student awareness and attitudes regarding pain management, which can help planners in the future to improve the quality of services for patients through better evaluation and treatment.

Several important limitations need to be considered, such as its small sample size, non‐probability sampling technique (a convenient sample) and use of self‐reporting. Also, using the English version of the tool may have impeded the students’ ability to understand some items. Though the findings of the current study might not be generalized for all Saudi universities (e.g. private universities), the findings provide a piece of beneficial evidence for academics and decision‐makers to integrate pain management education in different courses taught in nursing faculties.

Accordingly, we recommend further research studies to include a larger and more representative sample randomly selected from all Saudi universities (public and private) from different districts of the country. In addition, developing and applying an Arabic version of the KASRP questionnaire is highly recommended.
